# The role of globular heads of the C1q receptor in HPV 16 E2-induced human cervical squamous carcinoma cell apoptosis is associated with p38 MAPK/JNK activation

**DOI:** 10.1186/1479-5876-11-118

**Published:** 2013-05-08

**Authors:** Ling-juan Gao, Ping-qing Gu, Wei Zhao, Wen-yan Ding, Xue-qing Zhao, Shu-yu Guo, Tian-ying Zhong

**Affiliations:** 1 State Key Laboratory of Reproductive Medicine, Department of Clinical Laboratory, Nanjing Maternity and Child Health Care Hospital Affiliated to Nanjing Medical University, Tianfei Alley, Nanjing Mochou Road, Nanjing, 210004, P.R. China; 2 National Center for Epidemiology and Population Health, The Australian National University, Canberra, ACT, 0200, Australia

**Keywords:** Human papillomavirus type 16 (HPV 16) E2, Receptor for the globular heads of the human C1q (gC1qR), Apoptosis, Human cervical squamous carcinoma cells

## Abstract

**Background:**

Human papillomavirus type 16 (HPV 16) E2 protein is a multifunctional DNA-binding protein. HPV 16 E2 regulates many biological responses, including DNA replication, gene expression, and apoptosis. The purpose of this study was to investigate the relationship among the receptor for globular heads of the human C1q (gC1qR) gene expression, HPV 16 E2 transfection and apoptosis regulation in human cervical squamous carcinoma cells (C33a and SiHa).

**Methods:**

gC1qR expression was examined in C33a and SiHa cells using real-time PCR and Western blot analysis. Apoptosis of C33a and SiHa cells was assessed by flow cytometry. C33a and SiHa cell viability, migration and proliferation were detected using the water-soluble tetrazolium salt (WST-1) assay, a transwell assay and ^3^H-thymidine incorporation into DNA (^3^H-TdR), respectively.

**Results:**

C33a and SiHa cells that were transfected with a vector encoding HPV 16 E2 displayed significantly increased gC1qR gene expression and p38 mitogen-activated protein kinase (p38 MAPK)/ c-jun N-terminal kinase (JNK) activation as well as up-regulation of cellular apoptosis, which was abrogated by the addition of gC1qR small interfering RNA (siRNA). Furthermore, the changes in C33a and SiHa cell viability, migration and proliferation that were observed upon HPV 16 E2 transfection were abrogated by SB203580 (a p38 MAPK inhibitor) or SP600125 (a JNK inhibitor) treatment.

**Conclusion:**

These data support a mechanism whereby HPV 16 E2 induces apoptosis by silencing the gC1qR gene or inhibiting p38 MAPK/JNK signalling in cervical squamous cell carcinoma.

## Background

Persistent infection with a high-risk human papillomavirus (HPV) type has been correlated with the development of cervical cancer [1,2]. HPV 16 is responsible for over 50% of cervical cancer cases and is the second largest cause of cancer-related death in women worldwide, with an incidence of 500,000 malignancies per year, which includes carcinomas of the vagina, anus, vulva, penis and oropharynx [3,4]. The HPV 16 genome is composed of six regulatory proteins (E1, E2, E4, E5, E6, and E7) that regulate viral life cycle, gene expression, and cell function [5]. The HPV 16 E2 protein regulates viral DNA replication and transcription. The papillomavirus E2 protein is a 42-kDa nuclear protein containing two defined functional domains that are relatively conserved among papillomaviruses [6]. In addition to being a transcriptional regulator of HPV 16 E6 and E7 in early stages of the viral lifecycle, the E2 protein has potent antitumor activity in HPV 16-associated carcinogenesis [7]. HPV 16 E2 expression affects important cellular processes such as cellular proliferation or death, and loss of E2 gene integrity plays a role in the outcome and local control of cervical carcinomas [8,9].

Most HPV infections are eliminated through anti-viral immune responses, and only a percentage of HPV-infected women with oncogenic types have persistent infections that cause high-grade squamous intraepithelial lesions [10,11]. Although the immune response to cervical HPV infection is not well understood, recent cohort studies have highlighted that cervical HPV infection affects the maintenance of low cellular protein levels, changes viral protein expression and inhibits the host’s immune responses [12,13]. The complement system has been extensively characterised both biochemically and functionally. The receptor for the globular heads of C1q is gC1qR, a ubiquitous and highly anionic 33 kDa cellular protein that was initially identified as a mitochondrial matrix protein [14]. Indeed, gC1qR mediates many biological responses, including inflammation, infection and immune regulation [15]. Examples of such responses include phagocytosis and apoptotic cell uptake [16].

In the present study, our aim was to comprehensively identify cellular genes and biological processes that are regulated by HPV 16 E2. Our results provide evidence of an important role for the gC1qR gene in HPV 16 E2-induced apoptosis of C33a cells.

## Materials and methods

### Reagents

C33a and SiHa cervical squamous carcinoma cell lines were obtained from Hangzhou Hibio Bio-tech Co., Ltd (Hangzhou, Zhejiang, China). The Phototope-HRP Western Blot Detection System, including anti-mouse IgGs, HRP-linked antibodies, a biotinylated protein ladder, 20× LumiGLO Reagent and 20× peroxide, was purchased from Cell Signaling Technology (Beverly, MA, USA). The Annexin V-FITC/ Propidium Iodide (PI) Flow Cytometry Assay Kit was purchased from Invitrogen (Carlsbad, CA, USA). Antibodies directed against gC1qR, phosphorylated p38 MAPK (p*-p38 MAPK), phosphorylated JNK (p*-JNK), total p38 MAPK, total JNK and actin were purchased from Santa Cruz (Santa Cruz, CA, USA) and Cell Signaling Technology. pcDNA-HPV 16 E2 and pcDNA-HPV 16 E2 mutant (mut) plasmids were kindly supplied by Hangzhou Hibio Bio-tech Co., Ltd. gC1qR small interfering RNA (siRNA) and negative siRNA (siRNA directed toward an unrelated gene as a negative control) were synthesised by Wuhan Genesil Biotechnology Co., Ltd (Wuhan, China). Cell culture supplies were purchased from Life Technologies (Gaithersburg, MD, USA). Unless otherwise specified, all of the other reagents were of analytical grade.

### C33a And SiHa cell culture and DNA transfection conditions

C33a and SiHa cells were grown in Dulbecco’s modified Eagle medium (Gibco BRL, Grand Island, NY, USA), supplemented with 10% foetal bovine serum, 1% nonessential amino acids, and 2 mM glutamine. The cells were maintained in the presence of 5% CO_2_ at 37°C. Complementary DNA (cDNA) encoding HPV 16 E2 was cloned in frame using BamHI/EcoRI sites into the pcDNA 3.1 expression plasmid (Invitrogen, Carlsbad, CA). The resulting pcDNA-HPV 16 E2 vector was then transfected into C33a and SiHa cells. Twenty-four hours after plating, the cells were serum starved for an additional 24 h to quiescence. Following serum starvation, the cells were transfected using Lipofectamine^TM^ reagent (Life Technologies, Inc.) according to the vendor’s protocol. Briefly, 0.05-1.5 μg/ml plasmid DNA and 12 μg/ml Lipofectamine^TM^ were diluted in serum-free DMEM. After incubation for 30 min at 37°C, DNA-liposome complexes were added dropwise to each culture dish and incubated at 37°C in a 5% CO_2_ atmosphere for 12 h. Following transfection, the cells were cultured in serum-free DMEM. Reporter gene levels were normalised to total protein, and each experiment was independently performed three to five times.

### gC1qR SiRNA-expressing plasmid construction

We designed siRNA to target the 408–426 nucleotide portion of human gC1qR mRNA; the forward sequence was 5′-AAC AAC AGC AUC CCA CCA ACA UU-3′. A gC1qR siRNA-expressing plasmid was constructed using pGenesil-1 as the vector backbone. BamHI and HindIII restriction site overhangs were located near the 5′ end of the two oligonucleotides; a 6-nucleotide poly-T tract recognised as an RNA pol III termination signal was located at the 3′ end of the siRNA template. The siRNA was synthesised, annealed and ligated into the BamHI and HindIII restriction sites in the pGenesil-1 expression vector. A vector containing siRNA for an unrelated gene was used as a negative control.

### Real-time quantitative polymerase chain reaction (real-time qPCR)

Total RNA was isolated from tissue using Trizol reagent (Invitrogen, Carlsbad, CA, USA) according to the manufacturer’s instructions. Isolated RNA was then DNase-treated and reverse-transcribed according to the manufacturer’s instructions. To detect gC1qR expression, Primer-F (5′-AAT CAC ACG GTA GAC ACT GAA ATG CC-3′) and Primer-R (5′-CAT CAT CCC ATC TAA AAT GTC CCC TG-3′) were used with the FAM/TAMRA-labelled probe 5′-TGC TCC AGT TCA ACC AAC GTC CTT CTC-3′. β-actin was quantified using Primer-F (5′-TCA CCC ACA CTG TGC CCA TCT ATG A-3′) and Primer-R (5′-CAT CGG AAC CGC TCA TTG CCG ATA G-3′) with the FAM/TAMRA-labelled probe 5′-ACG CGC TCC CCC ATG CCA TCC TGC GT-3′. Quantitative real-time PCR was performed using an ABI PRISM 7300 sequence detection system with the following thermal cycling conditions: 2 min at 50°C and 10 min at 95°C followed by 40 cycles of 15 s at 95°C and 1 min at 60°C. All of the reactions were performed in 50 μL reaction volumes in triplicate. Standard curves were generated for gC1qR and β-actin. The β-actin gene was used as an internal control in all of the PCR experiments. The relative amounts of gC1qR mRNA were normalised to β-actin mRNA using the following formula: 2^− *ΔΔCT*^ = 2^− (*CT*. *gC*1*qR* − *CT*. *actin*)*Time* × + (*CT*. *gC*1*qR* − *CT*. *actin*)*Time* 0^.

### Western blot analysis

After various treatments, cells were harvested, pelleted by short centrifugation and suspended in lysis buffer (10 mM Tris–HCl, pH 7.8, 0.5% sodium deoxycholate, 0.5% Nonidet P-40, 100 mM NaCl, 10 mM EDTA) supplemented with protease inhibitors for 30 min on ice. The supernatants were collected by centrifugation at 13,000 × g at 4°C for 15 min. An equal amount of protein was separated by SDS-PAGE on a 10-15% polyacrylamide gel and transferred to a PVDF membrane. The transferred membranes were blocked for 1 h in 5% nonfat milk in PBST (PBS containing 0.05% Tween-20), incubated with appropriate primary antibodies followed by horseradish peroxidase-conjugated secondary antibodies. The protein bands were visualised using the enhanced chemiluminescence (ECL) Western Detection System.

### Cell viability analysis

The water-soluble tetrazolium salt (WST-1) assay (Roche Diagnostics, Mannheim, Germany) was performed to assess C33a and SiHa cell viability. The WST-1 assay is a colorimetric method in which the dye intensity is proportional to the number of viable cells. Cells were seeded into 96-well microtitre plates at a concentration of 5 × 10^3^ cells/well. After 12 h of incubation, cells were treated with for 48 h. After incubation, the cells were washed with PBS, WST-1 cell proliferation reagent was added, and the samples were incubated for 4 h. Sample absorbance was analysed with a bichromatic ELISA reader at 450 nm. All of the experiments were performed in triplicate with different C33a and SiHa cell passages.

### Cell migration analysis

C33a and SiHa cell (7.5 × 10^6^ cells/mL) migration was measured using 24 mm diameter chambers with 8 μm pore filters (Transwell, 6-well cell culture). Cells were collected and resuspended in serum-free media, and a 0.2 mL cell suspension was added to the upper chambers. Treatment media (0.5 mL) was added to the lower chambers. The chambers were incubated for 48 h at 37°C in a humidified atmosphere of 5% CO_2_/95% air. Next, the filters were fixed in 95% ethanol and stained with H&E. The upper filter surfaces were scraped twice with cotton swabs to remove non-migrated cells. Experiments were repeated in triplicate with different passages of the C33a and SiHa cells, and the migrated cells were counted microscopically (400 ×) in five different fields per filter.

### Apoptotic cell detection

C33a and SiHa cell apoptosis was detected using the Annexin V-FITC/propidium iodide (PI) staining kit via flow cytometry. After different treatments at the indicated times, C33a and SiHa cell were washed and resuspended in binding buffer (2.5 mM CaCl_2_, 10 mM HEPES, pH 7.4, and 140 mM NaCl) before being transferred to a 5 mL tube. The cells were incubated in the dark with 5 μL each of Annexin V-FITC and propidium iodide for 15 min. Binding buffer was then added to each tube, and the samples were analysed using a Beckman Coulter Epics XL flow cytometer. Annexin-V-FITC (−)/PI (−) staining indicated live cells, Annexin-V-FITC (+)/PI (−) staining indicated cells that were in the early stages of apoptosis, and Annexin-V-FITC (+)/PI (+) staining indicated cells that were in the late stages of apoptosis or necrosis.

### Statistical analysis

Most results are presented as the mean ± standard deviation (SD). Differences between data sets were assessed for significance using Student’s t-test, and a *p*-value less than 0.05 was considered significant (***p < 0.001; **p < 0.01; *p < 0.05; ^#^*p* > 0.05).

## Results

### The effect of HPV 16 E2 on cervical squamous carcinoma cell viability, migration and proliferation

**Figure 1:**
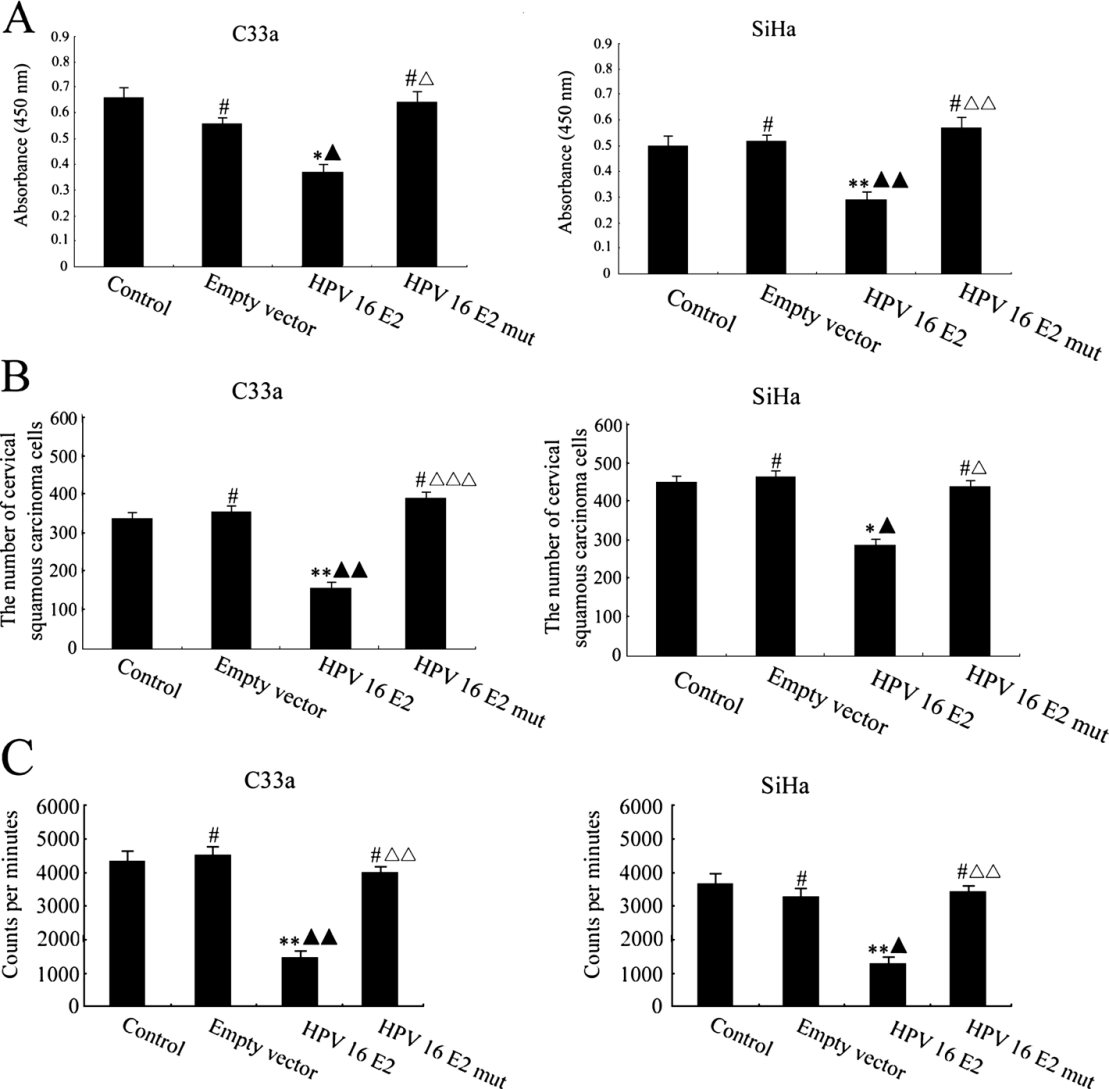
**The effect of HPV 16 E2 on cervical squamous carcinoma cell line (C33a and SiHa) viability, migration and proliferation.** Cells were treated with unmodified media (control), empty vector, HPV 16 E2 and HPV 16 E2 mutant for 48 h. **A:** C33a and SiHa cell viability was detected using a WST-1 assay. Sample absorbance was analysed using a bichromatic ELISA reader at 450 nm. **p < 0.01, *p < 0.05, ^#^*p* > 0.05 versus the control group; ^▲▲^*p* < 0.01, ^▲^*p* < 0.05 versus the empty vector group. ^△△^*p* < 0.01, ^△^*p* < 0.05 versus the HPV 16 E2 group. **B:** The C33a and SiHa cell migration was measured with a transwell assay. Migrated cells were counted microscopically (400 X) in five different fields per filter. **p < 0.01, *p < 0.05, ^#^*p* > 0.05 versus the control group; ^▲▲^*p* < 0.01, ^▲^*p* < 0.05 versus the empty vector group. ^△△△^*p* < 0.001, ^△^*p* < 0.05 versus the HPV 16 E2 group. **C:** C33a and SiHa cell proliferation. ^3^H-thymidine DNA incorporation results over 18 h of the final incubation. The results are expressed as the mean ± SD from 3 independent experiments. **p < 0.01, ^#^*p* > 0.05 versus the control group; ^▲▲^*p* < 0.01, ^▲^*p* < 0.05 versus the empty vector group; ^△△^*p* < 0.01 versus the HPV 16 E2 group.

To explore the effect of HPV 16 E2 on cervical squamous carcinoma cell viability, C33a and SiHa cells were assessed using a WST-1 assay following treatment with unmodified media (control group), empty vector, HPV 16 E2 and a HPV 16 E2 mutant. The data are presented in Figure 1A; HPV 16 E2 expression decreased cell viability compared with the unmodified media group, while there was no change in cell viability in the empty vector or HPV 16 E2 mutant group compared with the unmodified media group. Cell viability was notably decreased in cells transfected with the HPV 16 E2 vector compared with the empty vector group; moreover, cell viability was significantly different between the HPV 16 E2 and HPV 16 E2 mutant group.

The number of migrated cells was significantly lower in cells that were transfected with HPV 16 E2 compared with the unmodified media group. The number of migrated cells was not different among the empty vector group, the HPV 16 E2 mutant group and the unmodified media group (p > 0.05). Transfection of HPV 16 E2 significantly reduced the number of migrated cells compared with the empty vector group, whereas HPV 16 E2 mutant transfection significantly increased the number of migrated cells compared with the HPV 16 E2 vector group (Figure 1B).

As shown in Figure 1C, cervical squamous carcinoma cell DNA synthesis was lower in the HPV 16 E2 vector group than in the unmodified group. However, there was no difference in cell proliferation among the empty vector group, the HPV 16 E2 mutant group and the unmodified media group (p > 0.05). HPV 16 E2 vector transfection resulted in significantly reduced DNA synthesis in C33a and SiHa cells compared with the empty vector group, whereas HPV 16 E2 mutant transfection significantly increased the number of proliferating cells compared with the HPV 16 E2 vector group.

### The effect of HPV 16 E2 on gC1qR expression in cervical squamous carcinoma cells

**Figure 2:**
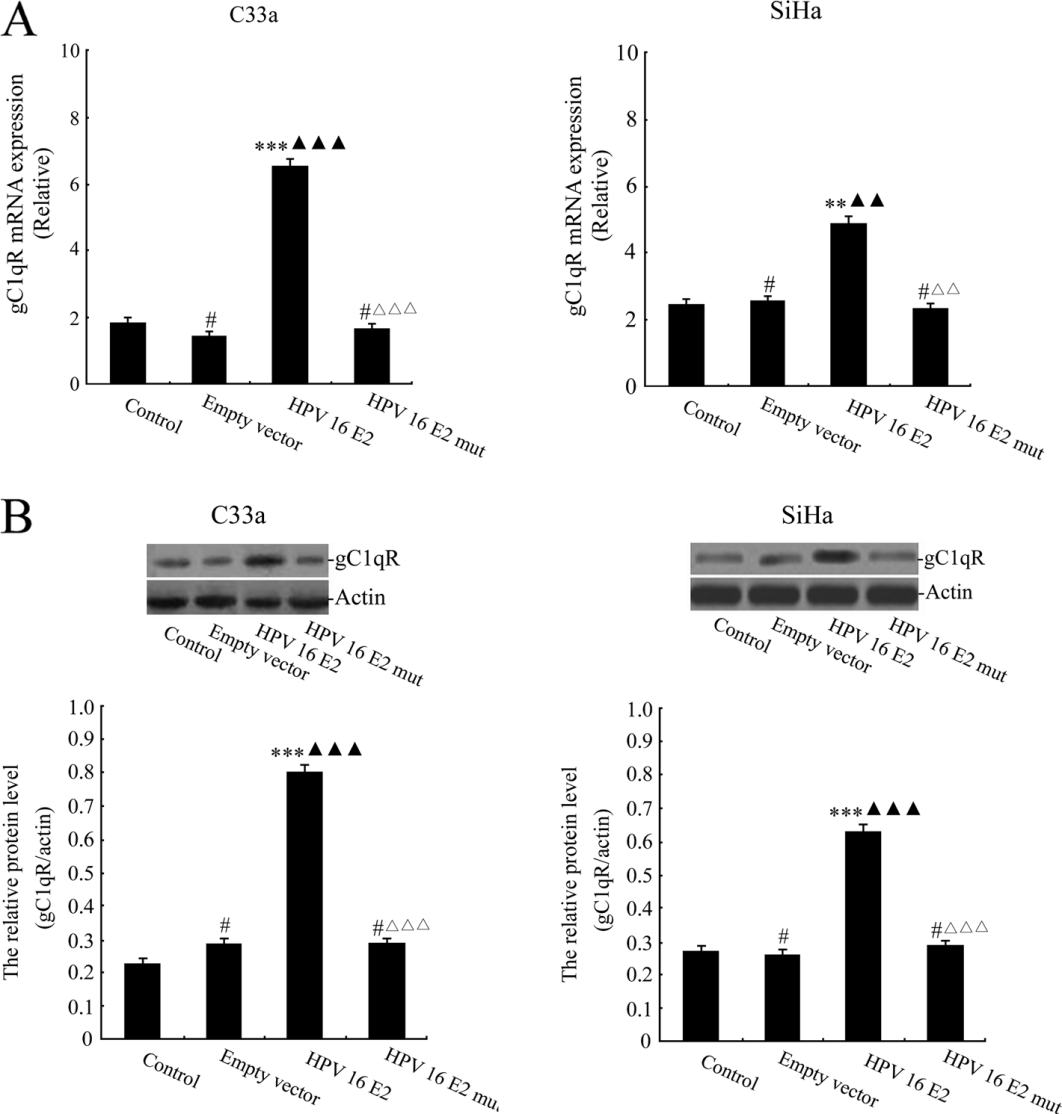
**The effect of HPV 16 E2 on gC1qR expression levels in cervical squamous carcinoma cell lines (C33a and SiHa).** Cells were treated with unmodified media (control), empty vector, HPV 16 E2 and HPV 16 E2 mutant for 48 h. **A:** Relative gC1qR gene expression levels are demonstrated in C33a and SiHa cells. gC1qR expression levels were analysed by real-time PCR. ***p < 0.001, **p < 0.01, ^#^*p* > 0.05 versus the control group; ^▲▲▲^*p* < 0.001, ^▲▲^*p* < 0.01 versus the empty vector group; ^△△△^*p* < 0.001, ^△△^*p* < 0.01 versus the HPV 16 E2 group. **B:** gC1qR protein levels were measured in C33a and SiHa cells using Western blot analysis. The graph depicts relative gC1qR protein levels normalised to actin. The results are expressed as the means ± SD of three separate experiments. ***p < 0.001, ^#^*p* > 0.05 versus the control group; ^▲▲▲^*p* < 0.001 versus the empty vector group; ^△△△^*p* < 0.001 versus the HPV 16 E2 group.

To investigate the effect of HPV 16 E2 on gC1qR expression in cervical squamous carcinoma cell lines, C33a and SiHa cells were treated with unmodified media (control group), empty vector, HPV 16 E2 and a HPV 16 E2 mutant. Real-time PCR and Western blot analysis results demonstrated that the gC1qR expression levels were significantly increased in the HPV 16 E2 group compared with the unmodified media and empty vector groups. However, gC1qR gene expression in the HPV 16 E2 mutant vector treated group was notably lower than that in the HPV 16 E2 vector group (Figure 2A-B). These findings suggest that HPV 16 E2 induces gC1qR gene expression.

### The effect of HPV 16 E2 on gC1qR expression, the p38 MAPK/ JNK signalling pathway and apoptosis in gC1qR-silenced cervical squamous carcinoma cells

**Figure 3:**
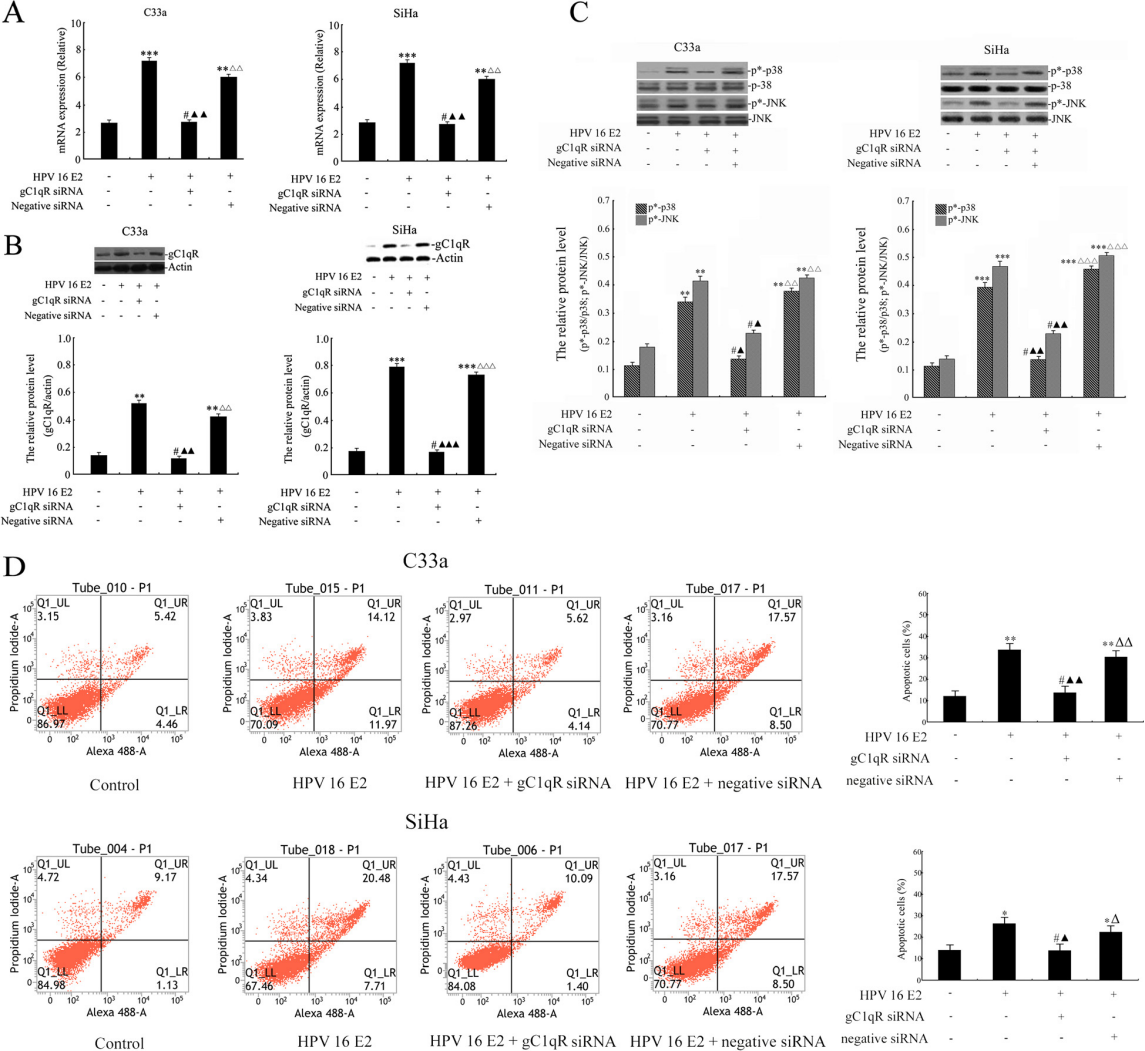
**The effect of HPV 16 E2 on gC1qR expression levels, the p38 MAPK/JNK signalling pathway and apoptosis of gC1qR-silenced cervical squamous carcinoma cell lines (C33a and SiHa).** Cells were treated with unmodified media (control) or HPV 16 E2 vector. After 72 h, the cells were transfected with 100 ng of gC1qR siRNA or 100 ng of negative siRNA. **A:** Relative gC1qR gene expression levels were analysed by real-time PCR. **B:** gC1qR protein levels were measured using Western blot analysis. The graph depicts the relative gC1qR protein levels normalised to actin. The results are expressed as the means ± SD of three separate experiments. **C:** Cells were lysed and examined for p*-p38 and p*-JNK by Western blot analysis. The graph shows relative phosphorylated p38 MAPK and JNK protein levels normalised to total p38 MAPK and JNK, respectively. The results are expressed as the means ± SD of three separate experiments. **D:** Numbers of apoptotic cells were measured by flow cytometry. The results are expressed as the means ± SD from 3 independent experiments. ***p < 0.001, **p < 0.01, *p < 0.05, ^#^*p* > 0.05 versus the HPV 16 E2 (−), gC1qR siRNA (−) and negative siRNA (−) groups; ^▲▲▲^*p* < 0.001, ^▲▲^*p* < 0.01, ^▲^*p* < 0.05 versus HPV 16 E2 (+), gC1qR siRNA (−) and negative siRNA (−) groups; ^△△△^*p* < 0.001, ^△△^*p* < 0.01, ^△^*p* < 0.05 versus HPV 16 E2 (+), gC1qR siRNA (+) and negative siRNA (−) groups.

To further explore the effect of HPV 16 E2 on gC1qR expression, the p38 MAPK/JNK signalling pathway and apoptosis in gC1qR-silenced cervical squamous carcinoma cells (see Additional file 1: Figure S1), C33a and SiHa cells were treated with unmodified media (control) or HPV 16 E2 vector. After 72 h, the cells were transfected with 100 ng of gC1qR siRNA or 100 ng of negative siRNA. gC1qR gene and protein expression levels were analysed by real-time PCR and Western blot analysis (Figure 3A-B). The results demonstrated that gC1qR mRNA and protein expression levels were significantly increased in the HPV 16 E2 vector group compared with the unmodified media group. However, there was no difference between the HPV 16 E2 + gC1qR siRNA group and the unmodified media group (p > 0.05). gC1qR expression in the HPV 16 E2 + gC1qR siRNA-treated group was significantly lower than that in the HPV 16 E2 group. In contrast, gC1qR expression was notably increased in the HPV 16 E2 + negative siRNA group compared with the HPV 16 E2 + gC1qR siRNA group.

Phospho-p38 MAPK and phospho-JNK were assessed by Western blot analysis in cervical squamous carcinoma cells that were treated with unmodified media (control) or HPV 16 E2 vector. After 72 h, the cells were transfected with 100 ng of gC1qR siRNA or 100 ng of negative siRNA. As shown in Figure 3C, phospho-p38 MAPK and phospho-JNK were significantly increased in the HPV 16 E2 group and the HPV 16 E2 + negative siRNA group compared with the unmodified media group. There was no difference in p*-p38 MAPK and p*-JNK protein expression between the unmodified media group and the HPV 16 E2 + gC1qR siRNA group. However, the phosphorylated p38 MAPK and JNK protein were notably lower in the HPV 16 E2 + gC1qR siRNA group compared with the HPV 16 E2 + negative siRNA group (Figure 3C).

C33a and SiHa cell apoptosis was assessed by flow cytometry following treatment with unmodified media (control) or HPV 16 E2 vector. After 72 h, the cells were transfected with 100 ng of gC1qR siRNA or 100 ng of negative siRNA. The cells were double-stained with Annexin V and PI. Early and late apoptotic cells were distributed in the Q1_LR and Q1_UR regions, respectively. Necrotic cells were located in the Q1_UL region. Figure 3D shows that accumulated HPV 16 E2 increased the C33a and SiHa cell number in the Q1_LR and Q1_UR regions in the HPV 16 E2 vector group and the HPV 16 E2 + negative siRNA group compared with the unmodified media group. However, the Q1_LR and Q1_UR regions in the HPV 16 E2 + gC1qR siRNA vector-transfected cells showed a notable decrease compared with the HPV 16 E2 vector-transfected group. However, the apoptotic cells in the HPV 16 E2 + gC1qR siRNA group were significantly decreased compared with the HPV 16 E2 + negative siRNA group.

### The effect of HPV 16 E2 combined with SB203580 or SP600125 on gC1qR expression and cervical squamous carcinoma cell viability, migration and proliferation

**Figure 4:**
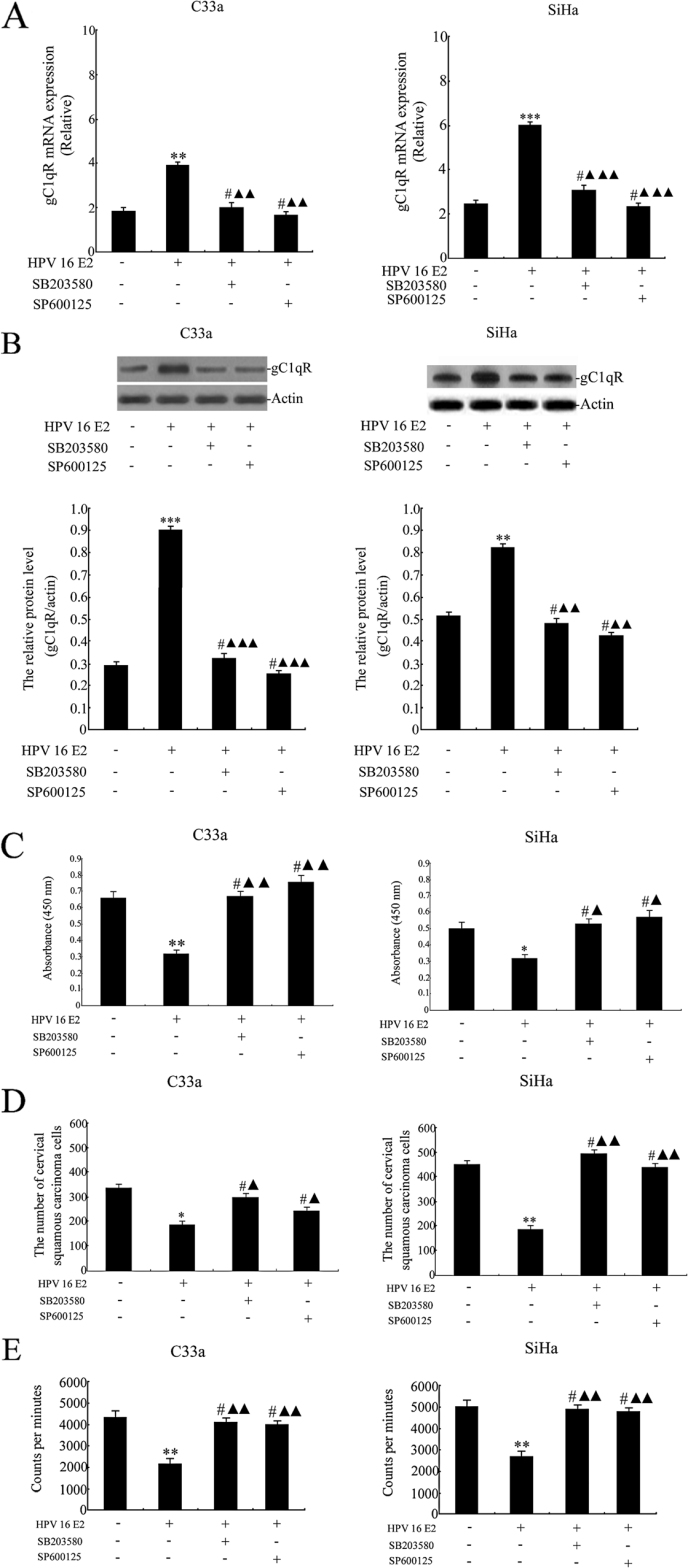
**The effect of HPV 16 E2 combined with SB203580 or SP600125 on gC1qR expression, and cervical squamous carcinoma cell line (C33a and SiHa) viability, migration and proliferation.** The cells were treated with unmodified media (control) or HPV 16 E2 vector. After 72 h, the cells were treated with 20 μM SB203580 (a p38 MAPK pathway inhibitor) or 30 μM SP600125 (a JNK pathway inhibitor). **A:** Relative gC1qR gene expression levels are shown in C33a and SiHa cervical squamous carcinoma cell lines. gC1qR expression levels were analysed by real-time PCR. **B:** gC1qR protein expression levels were measured in C33a and SiHa cells using Western blot analysis. The graph depicts relative gC1qR protein levels normalised to actin. The results are expressed as the means ± SD of three separate experiments. **C:** C33a and SiHa cell viability was detected using a WST-1 assay. Sample absorbance was analysed using a bichromatic ELISA reader at 450 nm. **D:** C33a and SiHa cell migration was measured by a transwell assay. Migrated cells were counted via microscopy (400 X) in five different fields per filter. **E:** C33a and SiHa cell proliferation. ^3^H-thymidine DNA incorporation over the last 18 h of the final incubation. The results are expressed as the mean ± SD from 3 independent experiments. ***p < 0.001, **p < 0.01, *p < 0.05, ^#^*p* > 0.05 versus the HPV 16 E2 (−), SB203580 (−), and SP600125 (−) groups; ^▲▲▲^*p* < 0.001, ^▲▲^*p* < 0.01, ^▲^*p* < 0.05 versus the HPV 16 E2 (+), SB203580 (−), and SP600125 (−) groups.

In these experiments, C33a and SiHa cells were treated with unmodified media (control) or HPV 16 E2 vector. After 72 h, the cells were treated with 20 μM SB203580 (a p38 MAPK pathway inhibitor) or 30 μM SP600125 (a JNK pathway inhibitor). gC1qR gene and protein expression levels were analysed by real-time PCR and Western blot analysis (Figure 4A-B). The results demonstrated that the gC1qR mRNA and protein levels were significantly increased in the HPV 16 E2 vector group compared with the unmodified media group. However, there were no differences among the HPV 16 E2 + SB203580 group, the HPV 16 E2 + SP600125 group and the unmodified media groups (p > 0.05). In contrast, gC1qR expression in the HPV 16 E2 + SB203580 group and the HPV 16 E2 + SP600125 group was notably reduced compared with the HPV 16 E2 group.

To explore the effect of HPV 16 E2 combined with SB203580 or SP600125 on cervical squamous carcinoma cells viability, cells were treated with unmodified media (control) or HPV 16 E2 vector. After 72 h, the cells were treated with 20 μM SB203580 (a p38 MAPK pathway inhibitor) or 30 μM SP600125 (a JNK pathway inhibitor). The results demonstrated that HPV 16 E2 decreased cell viability compared with the unmodified media group. However, cell viability in the HPV 16 E2 + SB203580 group and the HPV 16 E2 + SP600125 group was not changed compared with the unmodified media group. Cell viability was notably increased in cells that were treated with HPV 16 E2 + SB203580 or HPV 16 E2 + SP600125 compared with the HPV 16 E2 vector group (Figure 4C).

HPV 16 E2 transfection caused a significant repression of migrated cells that was comparable to the unmodified media group. However, the number of migrated cells was not different among the HPV 16 E2 + SB203580 group, the HPV 16 E2 + SP600125 group and the unmodified media group (p > 0.05). Transfection of HPV 16 E2 + SB203580 or HPV 16 E2 + SP600125 significantly increased the number of migrated cells compared with the HPV 16 E2 vector group (Figure 4D).

As shown in Figure 4E, C33a and SiHa cell proliferation was significantly decreased in HPV 16 E2-transfected cells compared with the unmodified media group. The numbers of proliferating cells were not different among the HPV 16 E2 + SB203580 group, the HPV 16 E2 + SP600125 group and the unmodified media group (p > 0.05). Interestingly, transfection of HPV 16 E2 + SB203580 or HPV 16 E2 + SP600125 increased cell proliferation compared with the HPV 16 E2 vector group.

## Discussion

In the present study, we identified gC1qR as a downstream target for p38 MAPK/JNK signalling in HPV 16 E2-induced cervical squamous carcinoma cell apoptosis. Our analysis provided experimental evidence that silencing the gC1qR gene or inhibiting p38 MAPK/JNK signalling is essential for the *in vitro* growth and migration properties of cervical squamous carcinoma cells in response to HPV 16 E2 treatment.

The C33a cell line was the primary focus of this experiment because C33a cells are negative for HPV DNA and RNA [17], and they represent a convenient model to study the effects of HPV 16 E2 on cellular gene expression without the involvement of other HPV types. Traditionally, the effects observed in regulating cellular genes when E2 protein is expressed in cervical carcinoma-derived cell lines result from repressed expression of the viral oncogenes E6 and E7; however, in this work, we demonstrated that HPV16 E2 changes cellular gene expression independently of viral oncoprotein E6 and E7 regulation.

HPV type 16 is the most prevalent type of HPV (84.7%), which is in agreement with other studies, while the frequency of HPV type 18 (3.38%) is very low compared with other ethnic populations [18]. Low-grade dysplasias with HPV 16 infection demonstrated an increased rate of malignancy progression [19]. HPV-16 E6/E7 oncoproteins have been demonstrated to cause immortalisation of primary human keratinocytes and are expressed in malignant cancers [20]. Many studies have previously reported the ability of the HPV-16 E6/E7 oncoproteins to disrupt the normal process of differentiation of human foreskin keratinocytes [21] by targeting key tumour suppressors, such as p53 [22] and pRb [23], resulting in increased levels of cell survival proteins, such as Akt [24], and disruption of the cell cycle [25]. The HPV E2 protein functions as a repressor or an activator of early gene transcription, which regulates viral transcription and genome replication [26]. Disruption of the viral E2-gene, which controls the transcription of oncogenes E6 and E7 that manipulate the cell cycle and the ability of apoptosis, has been associated with poor outcomes. Conversely, the HPV 16 E2 gene acted via mitochondrial-dependent pathways to control cellular apoptosis and fate [27]. Among mitochondrial matrix proteins, gC1qR controls diverse cellular processes, such as cell growth, differentiation and apoptosis [28]. The present study provides an essential framework for assessing the role of gC1qR protein in HPV 16 E2-transfected cervical squamous carcinoma cell apoptosis. gC1qR is a multi-compartmental and multi-functional cellular protein that is distributed in several tissues and cell types, including lymphocytes, endothelial cells, dendritic cells and platelets [29,30]. However, in our experiment, immunohistochemistry demonstrated that gC1qR expression was significantly decreased in human cervical squamous cell carcinoma tissues compared with normal cervical tissues (see Additional file 2: Figure S2). Although gC1qR is not overexpressed in human cervical squamous cell carcinoma tissues, its expression increased significantly in the HPV16 E2-induced cervical squamous carcinoma cell line.

During complement activation, the biological responses mediated by C1q recognise and activate the signal that triggers the classical complement pathway. C1q functions as a potent extracellular signal for a wide range of cells, resulting in inhibition of T cell proliferation or endothelial cell activation [31]. Additionally, the C1q-gC1qR complex not only may be involved in innate and adaptive immunity [32], but also may be an underlying molecular mechanism in virus infection. Xu et al. [33] provided evidence that viruses use host gC1qR protein to inhibit antiviral responses and to promote viral proliferation by activating a suppressive pathway to negatively regulate antiviral signalling. When constitutively expressed in a normal murine fibroblast cell line, gC1qR induces growth perturbation, morphological abnormalities and apoptosis [34]. gC1qR has been extensively studied previously as an inducer of apoptosis [35]. Recent cohort studies have shown that gC1qR is a conserved eukaryotic multifunctional protein that primarily localised in the mitochondrial matrix (see Additional file 3: Figure S3) and on the cell surface [36]. Human gC1qR is expressed as a proprotein of 282 amino acids (aa) whose first 73 amino acids, containing a mitochondrial localization signal, are required for localizing the protein to the mitochondria and are subsequently cleaved to generate mature gC1qR. The mature form of gC1qR has been tied to apoptosis and autophagy via inducing mitochondrial dysfunction [37]. In the present study, we determined that silencing the gC1qR gene in cervical squamous carcinoma cells results in decreased cervical squamous carcinoma cell apoptosis rates.

In the present study, our results indicate that gC1qR is a physiological inhibitor of HPV 16-induced cervical squamous carcinoma cell survival. A role for gC1qR in HPV 16 E2 oncogene-mediated apoptosis was also demonstrated. As shown in Figure 3D, flow cytometry analysis revealed that cells in the subG1 region decreased after gC1qR siRNA vector treatment. Interestingly, we observed that the gC1qR gene has an effect on the p38 MAPK/JNK-pathway in HPV 16 E2 expression. Recently, it was reported that the p38 MAPK/JNK-pathway is activated by HPV 16 E6 and E7 viral oncogene expression [38]. However, our observations suggest that HPV 16 E2 also activates this pathway; however, the consequences of this activation may be different from the activation induced by the viral oncogenes because tight regulation and controlled coordination of the p38 MAPK/JNK signalling cascade is required to maintain the balance between apoptosis and differentiation.

## Conclusion

In this work, our results demonstrate that HPV 16 E2 regulates cellular gene expression independently of the viral oncoproteins E6 and E7. The data presented in this study demonstrate that E2 predominantly up-regulates gC1qR gene expression, which induces cervical cancer cell apoptosis. The expression of HPV 16 E2 by cells suggests that increased gC1qR levels are important in cervical squamous carcinoma cell apoptosis and that gC1qR induces apoptosis through the p38 MAPK/JNK signalling pathway in human cervical squamous carcinoma cells.

## Abbreviations

HPV 16: Human papillomavirus type 16; gC1qR: globular heads of C1q receptor; 3H-TdR assay: ^3^H-thymidine incorporation into DNA; p38 MAPK: p38 mitogen-activated protein kinase; JNK: c-jun N-terminal kinase; siRNA: small interfering RNA; SOCS: suppressor of cytokine signalling; PI: Propidium iodide; cDNA: Complementary DNA; WST-1: water-soluble tetrazolium salt; SD: standard deviation; real-time PCR: real-time quantitative polymerase chain reaction; mt: mutant.

## Competing interests

The authors declare that they have no competing interests.

## Authors’ contributions

TYZ conceived of the study and drafted the manuscript. GLJ participated in its design and helped draft the manuscript. PQG and WZ performed the molecular biological studies and the statistical analysis. WYD collected patient information. XQZ helped revise the manuscript and performed statistical analysis. All of the authors read and approved the final manuscript.

## Supplementary Material

Additional file 1: Figure S1 The levels of gC1qR expression. C33a and SiHa cells were treated with plain medium (control), negative siRNA and gC1qR siRNA for 48 h. The expression of the gC1qR protein was measured by Western blot analysis. The graph depicts the relative gC1qR protein levels normalised to actin. The results are expressed as the mean ± SD of three separate experiments. ^*^p < 0.05, ^#^*p* > 0.05 versus the plain medium group. Click here for file

Additional file 2: Figure S2 The results of immunohistochemical staining. The positive results for gC1qR antigen in human cervical tissues by immunohistochemistry (× 200). **A:** staining of monoclonal anti-gC1qR antibody in human cervical squamous cells carcinoma tissues; **B:** staining of monoclonal anti-gC1qR antibody in normal cervix tissues. Click here for file

Additional file 3: Figure S3 The subcellular localization of gC1qR protein in C33a cells. In this experiment, the intracellular localisation of gC1qR was detected by cellular fractionation. The C33a cells were separated into endoplasmic reticulum (ER), nuclei (Nu), and mitochondrial (Mt) fractions. Calnexin, histone H1 and mtSSB were detected by western blotting as markers for endoplasmic reticulum, nuclei and mitochondria, respectively. The expression of gC1qR protein was detected in endoplasmic reticulum (ER), nuclei (Nu) and mitochondria (Mt) in C33a cells. The expression of gC1qR protein was localised to the mitochondrial fraction. Click here for file
