# Supplementary figures and images for: The role of globular heads of the C1q receptor in HPV 16 E2-induced human cervical squamous carcinoma cell apoptosis is associated with p38 MAPK/JNK activation
Source: J Transl Med. 2013 May 8;11:118. doi: 10.1186/1479-5876-11-118 (PMC3651870; doi:10.1186/1479-5876-11-118)

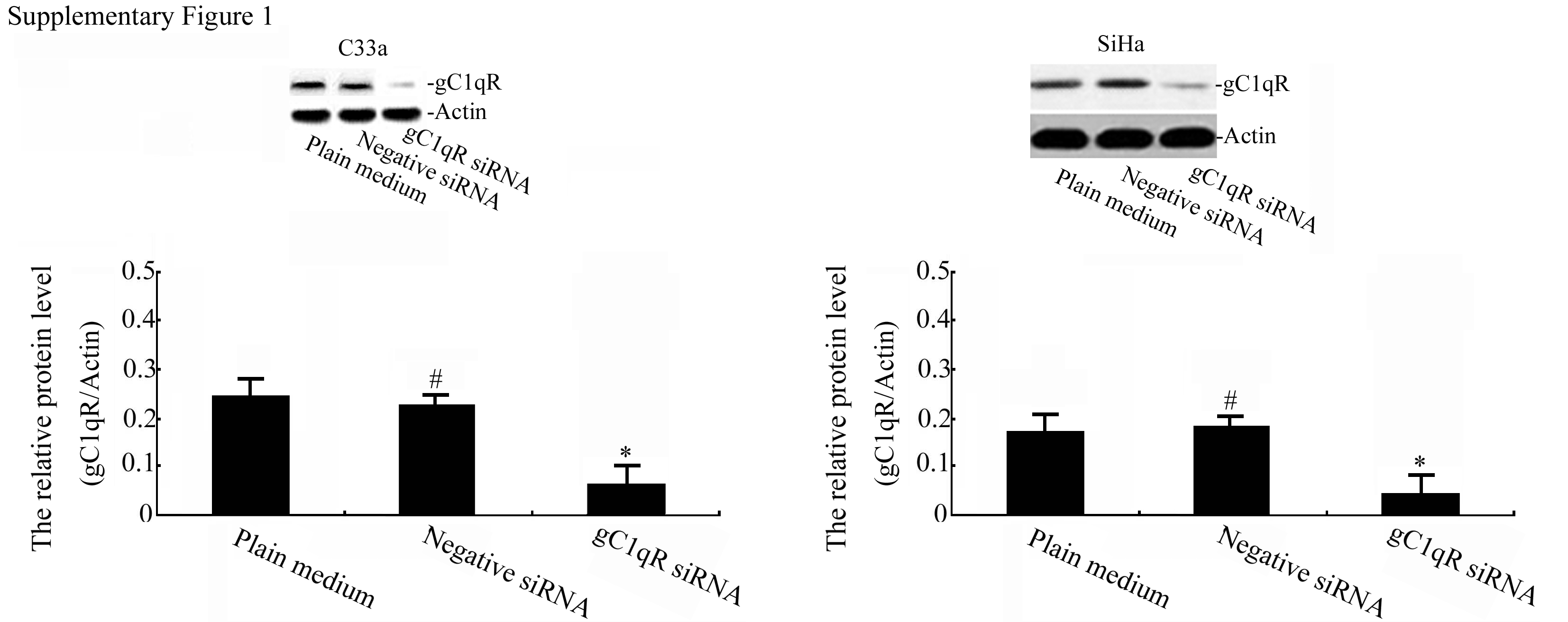

Supplement: Additional file 1: Figure S1 — The levels of gC1qR expression. C33a and SiHa cells were treated with plain medium (control), negative siRNA and gC1qR siRNA for 48 h. The expression of the gC1qR protein was measured by Western blot analysis. The graph depicts the relative gC1qR protein levels normalised to actin. The results are expressed as the mean ± SD of three separate experiments. *p < 0.05, #p > 0.05 versus the plain medium group. [file 1479-5876-11-118-S1.tiff]

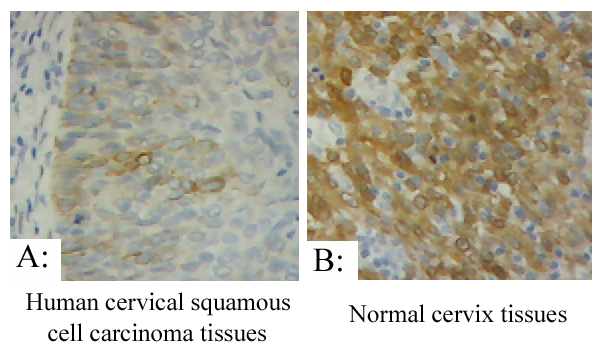

Supplement: Additional file 2: Figure S2 — The results of immunohistochemical staining. The positive results for gC1qR antigen in human cervical tissues by immunohistochemistry (× 200). A: staining of monoclonal anti-gC1qR antibody in human cervical squamous cells carcinoma tissues; B: staining of monoclonal anti-gC1qR antibody in normal cervix tissues. [file 1479-5876-11-118-S2.tiff]

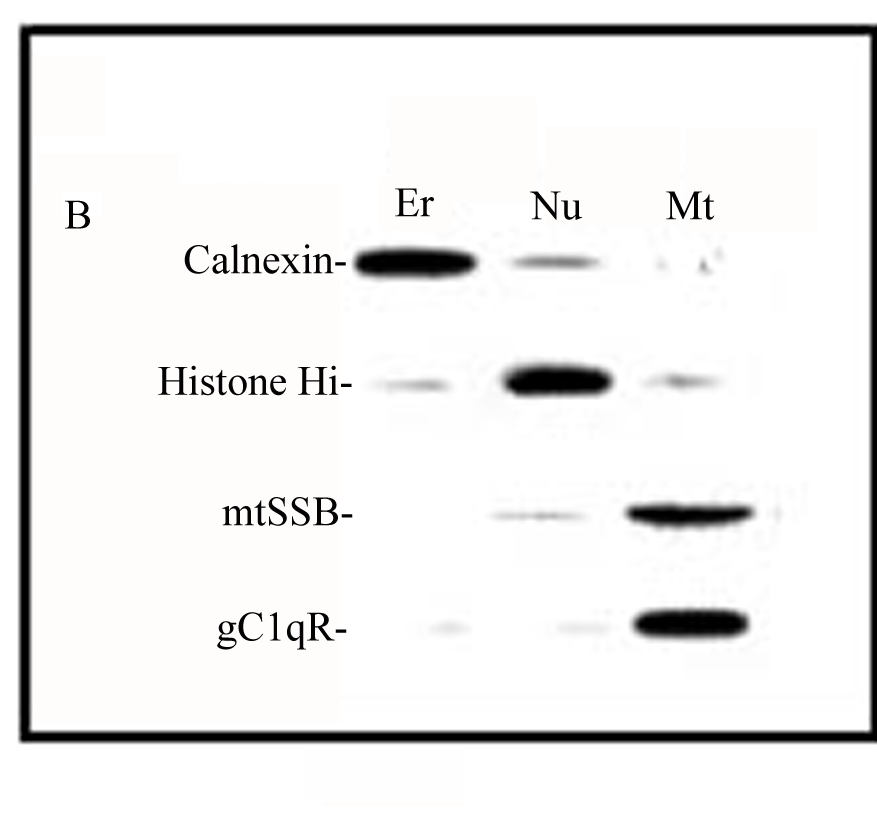

Supplement: Additional file 3: Figure S3 — The subcellular localization of gC1qR protein in C33a cells. In this experiment, the intracellular localisation of gC1qR was detected by cellular fractionation. The C33a cells were separated into endoplasmic reticulum (ER), nuclei (Nu), and mitochondrial (Mt) fractions. Calnexin, histone H1 and mtSSB were detected by western blotting as markers for endoplasmic reticulum, nuclei and mitochondria, respectively. The expression of gC1qR protein was detected in endoplasmic reticulum (ER), nuclei (Nu) and mitochondria (Mt) in C33a cells. The expression of gC1qR protein was localised to the mitochondrial fraction. [file 1479-5876-11-118-S3.tiff]
